# Health economic analysis of antiviral drugs in the global polio eradication endgame

**DOI:** 10.1177/0272989X231191127

**Published:** 2023-08-14

**Authors:** Kamran Badizadegan, Dominika A. Kalkowska, Kimberly M. Thompson

**Affiliations:** 1 Kid Risk, Inc., Orlando, FL 32819, USA

**Keywords:** Polio, vaccines, antiviral drugs, modeling, disease eradication, prevention

## Abstract

**Background:**

Pollio antiviral drugs (PAVDs) may provide a critical tool in the eradication endgame by stopping poliovirus infections in immunodeficient individuals who may not clear the virus without therapeutic intervention. Although prolonged/chronic poliovirus excreters are rare, they represent a source of poliovirus reintroduction into general population. Prior studies that assumed successful cessation of all oral poliovirus vaccine (OPV) use estimated the potential upper bound of the incremental net benefits (INBs) of resource investments in research and development of PAVDs. However, delays in polio eradication, OPV cessation, and the development of PAVDs necessitate an updated economic analysis to reevaluate the costs and benefits of further investments in PAVDs.

**Methods:**

Using a global integrated model of polio transmission, immunity, vaccine dynamics, risks, and economics, we explore the risks of reintroduction of polio transmission due to immunodeficiency-related vaccine derived poliovirus (iVDPV) excreters and reevaluate the upper bound of the INBs of PAVDs.

**Results:**

Under the current conditions, for which the use of OPV will likely continue for the foreseeable future, even with successful eradication of type 1 WPV by the end of 2023 and continued use of Sabin OPV for outbreak response, we estimate upper bound INB of 60 million US$2019. With >100 million US$2019 already invested in PAVD development and with the introduction of novel OPVs that are less likely to revert to neurovirulence, our analysis suggests the expected INBs of PAVDs would not offset their costs.

**Conclusions:**

While PAVDs could play an important role in the polio endgame, their expected economic benefits drop with ongoing OPV use and poliovirus transmissions. However, stakeholders may pursue development of PAVDs as a desired product regardless of their economic benefits.

## 1. Background

Primary immunodeficiencies (PIDs), now formally grouped under Inborn Errors of Immunity (IEI),^1,2^ may pose challenges to global infectious disease management and eradication efforts. Specifically, individuals with PID may not clear an acquired infection or an inoculated live vaccine, thus making them a potential long-term reservoir for reintroduction of poliovirus after eradication of indigenous strains. As the Global Polio Eradication Initiative (GPEI) has progressed through the polio endgame, it has increasingly recognized the persistent shedding of oral poliovirus vaccines (OPVs) by individuals with PIDs as a risk for the reintroduction of potentially pathogenic polioviruses into the polio-free populations.^3,4^

Reporting of persistent OPV shedding by an immunocompromised patient first appeared shortly after introduction of OPV use in the 1960s.^5^ Subsequent observations suggested the possibility of reversion of OPV to the wild phenotype in immunodeficient individuals receiving OPV, as demonstrated by a 3-year-old boy with agammaglobulinemia.^6^ Soon thereafter, reports of vaccine-associated paralytic polio (VAPP) and vaccine-derived polioviruses (VDPVs) began to emerge, preferentially involving individuals with PIDs.^7–14^ The pathophysiology of VAPP is somewhat uncertain with VAPP largely considered an adverse individual reaction. On the other hand, VDPVs are of great public health concern as they represent the ability of OPV to accumulate enough genetic alterations following persistent replication to behave phenotypically like a wild poliovirus (WPV).^3,15^

Poliovirus genomes evolve at a rate of ~1% per year with sustained transmission.^16^ In communities that use OPV with low coverage, persistent secondary and community transmission can lead to loss of attenuating mutations and consequent outbreaks of circulating VDPVs (cVDPVs) that clinically behave like the homotypic WPVs.^3,17,18^ Similarly, when immunodeficient individuals become prolonged or chronic excreters (and hence replicators) of live polioviruses following inoculation with an OPV or infection with a live poliovirus, they can potentially reintroduce the transmission of a pathogenic immunodeficiency-related vaccine derived poliovirus (iVDPV) into the population.^3,19,20^ Reintroduction of live poliovirus transmission by an iVDPV excreter into a previously polio-free region has not been documented to date, but case reports from the US,^21^ Philippines,^22^ and Israel^23^ suggest the possibility, and the risk remains as long as OPVs remain in use. Newly-developed novel OPVs (nOPVs)^24,25^ may similarly evolve.^26^ However, early evidence from use of a genetically-modified novel type 2 OPV (nOPV2) under emergency use licensure (EUL)^24^ suggests lower the risk of reversion to a pathogenic phenotype.^27^ Clinical and genetic behavior of nOPV2 in individuals with PIDs is currently unknown.

Despite earlier commitments to end all use of OPV and fully contain all live polioviruses,^28^ and to substantially reduce OPV-related reintroduction risks,^3,29^ the phased cessation of type 2-containing OPV (OPV2) in 2016^30^ did not end all reported type 2 cases or OPV2 use.^31–34^ In addition, GPEI plans related to completing OPV cessation for types 1 and 3 remain uncertain,^35^ making it difficult to prospectively model the polio endgame and the associated health economic outcomes of specific interventions. The ongoing challenges with OPV cessation motivated the creation of a GPEI bOPV cessation team.^36^ The 2022–2026 GPEI strategic plan aims to eradicate both WPV1 and cVDPV2 transmission by the end of 2023, to certify these achievements by 2026, and to coordinate the global cessation of the use of bivalent OPV (bOPV, containing types 1 and 3 OPV) in 2027.^35^ Recent polio endgame modeling that assumed insufficient population immunity to stop and prevent cVDPVs for types 1 and 2 prior to bOPV cessation in 2027 (representing the current world trajectory), anticipated increasing cases of polio after 2027.^37^

With substantial financial requirements for GPEI,^35^ decreasing expected incremental net benefits of polio eradication with continued delays,^38^ and increasing expectations for health services integration,^39^ budgetary pressures will likely motivate GPEI partners to prioritize resources toward the programmatic activities expected to yield the greatest returns. Any investments in expensive polio-related risk management interventions represent key areas for health economic evaluation to inform investment decisions.

Many prior studies published 2000–2019^40^ and since then^38,41,42^ characterized expected financial costs and benefits of polio interventions. Some prior health economic studies specifically considered the potential role of polio antiviral drugs (PAVDs) and screening to identify iVDPV excreters.^19,43^ The framing of prior analyses related to the incremental net benefits (INBs) of PAVDs focused on providing a bounding estimate of the potential benefits of both finding (through screening) and treating iVDPV excreters with PAVDs.^19,43^ The first analysis estimated an upper bound of $0.5 billion (US$2013).^19^ The second analysis estimated potential benefits of $0.26 to $1.5 billion 2013 US dollars (US$2013), with the range depending on the reintroduction risks posed by iVDPVs, effectiveness of the PAVD, and ability of the screening efforts to identify asymptomatic iVDPV excreters.^43^ Now with more than a decade of experience in PAVDs development,^44–47^ and with a new landscape for the polio endgame^37^ and the associated health economics,^38,48^ we update the estimates of the INBs for PAVDs. The results presented here should help guide investment decisions by the GPEI funding partners for the prospective polio endgame.

## 2. Methods

### Effectiveness of interventions for iVDPVs

**Figure 1:**
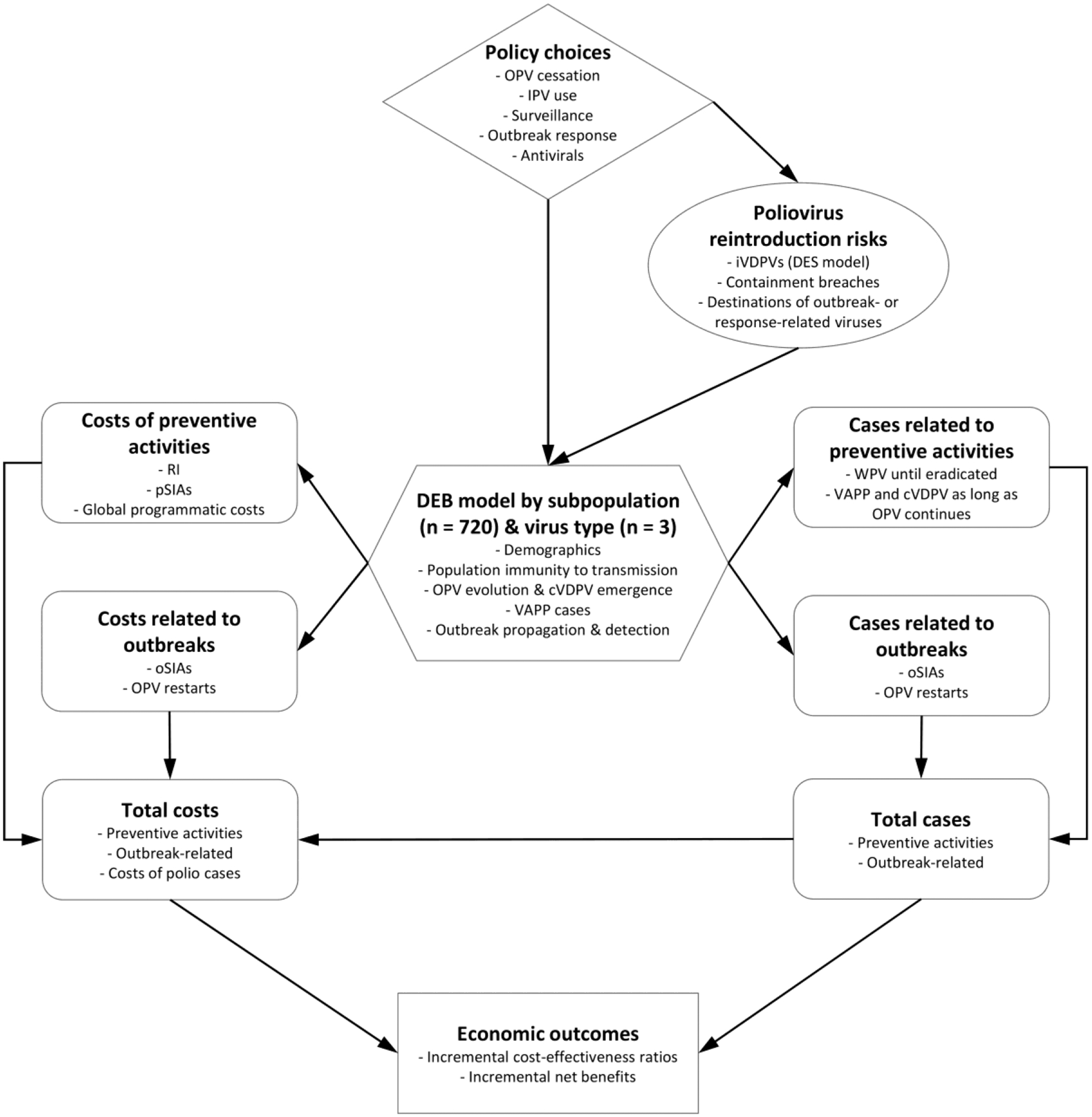
Schematic of components of the integrated global model **Abbreviations**: cVDPV, circulating VDPV; DEB, differential equation-based model; DES, discrete-event simulation model; iVDPV, immunodeficiency-associated vaccine-derived poliovirus; IPV, inactivated poliovirus vaccine; OPV, oral poliovirus vaccine; oSIAs, outbreak supplemental immunization activities; PAVD, polio antiviral drug; pSIAs, preventive supplemental immunization activity; VAPP, vaccine-associated paralytic polio; VDPV, vaccine-derived poliovirus; WPV, wild poliovirus

We use an integrated poliovirus model that includes numerous interrelated components^29,49,50^ (see Supplement for details). We require an integrated model for this analysis to simultaneously deal with the rare events of iVDPVs at the individual level and consequences at stochastic reintroductions of polioviruses into populations aggregated to the global level, and to account for global variability in risks, vaccine use, and poliovirus transmissibility. Figure 1 provides an overall schematic of the components of the integrated model.^47^ At its core, the integrated model relies on a differential equation-based (DEB) poliovirus transmission and OPV evolution model, that tracks transmission of each of the 3 types of polioviruses independently and the dynamics of immunity and infections for 8 different immunity states and waning that account for differential abilities to become infected (or reinfected) and participate in transmission.^49^ The DEB further characterizes infection using multiple stages and the potential evolution of OPV to a cVDPV that behaves like homotypic wild poliovirus using 20 stages, with the input assumptions for the DEB and OPV evolution transmission model calibrated using epidemiological experience for a wide range of situations.^49^

Specifically important for iVDPV risks, we apply a discrete-event simulation model (DES) component that characterizes long-term iVDPV excreter prevalence as a function of the timing of OPV cessation for each poliovirus serotype and other inputs.^19,20,43,47^ The DES tracks PID patients progressing through various clinical and OPV infection stages using a discrete time step of 1 month. The DES uses the same assumptions as the DEB model to account for different characteristics of transmission and vaccine schedules when stratifying the global population into blocks and subpopulations.^29,49,50^ Specifically, all components in the integrated model stratifies the world into 72 epidemiological blocks of 10 subpopulation of approximately 10.7 million people each, with each subpopulation assigned a World Bank Income Level (low-income, LI; lower middle-income, LMI; upper middle-income, UMI; high-income, HI) and current vaccine use in routine immunization (RI, i.e., OPV+IPV, IPV/OPV, IPV-only) that abstractly represent variability in the global population. The model assigns demographics and a basic reproduction number (R_0_) for each subpopulation to account for many factors that affect poliovirus transmission and health system quality, with the same inputs used for all components shown in Figure 1.^29,49,50^ The DES model assigns attributes at birth for individuals modeled as PIDs, including monthly event probabilities (i.e., PID onset, diagnosis, treatment, OPV infections, VAPP in an immunodeficient individual (iVAPP), mortality).^20^ The integrated model accounts for critical feedback loops, particularly highlighting the role of any ongoing use of (and thus potential ongoing exposure to) OPV.^47^

To evaluate the expected value of the INBs of investments in PAVDs as a function of different levels of PAVD effectiveness and approaches for screening to identify iVDPV excreters, the integrated model simulates 100 stochastic iterations that introduce prospective reintroduction risks related to iVDPVs, containment breaches, and other risks that can restart transmission in modeled populations.^20,50^ Notably, each stochastic iteration of the integrated model uses a corresponding stochastic realization of the DES model to create random potential iVDPV introductions into the general population and randomly generated contacts with the general population for each active long-term iVDPV excreter after type-specific OPV cessation.^20,50^ We assume that iVDPVs enter the general population at OPV reversion stage 10 (i.e., midway in the 20-stage OPV evolution process for each type of poliovirus that begins with the behavior of Sabin OPV and evolves to behave like a fully-reverted or wild poliovirus).^20,49,50^ We assume that exposure to iVDPVs in the general population may or may not lead to effective introductions (i.e., reestablishing transmission).

The integrated model^50^ also uses the DES model to create new iVDPV excreters as a result of any post-OPV cessation outbreak response use of OPV, by creating a list of individuals born with a pre-disposition of developing a PID with an iVDPV excreter potential acquisition status. In case of post-OPV cessation OPV use for outbreak response, the integrated model calculates the probability of infection given exposure for each alive, clinical, not-yet-infected PID patient (based on pre-determined PID events and infection probabilities) that may or may not lead to new infections. We assume that the use of Sabin monovalent OPV (mOPV) for outbreak response would lead to the highest potential number of new iVDPV excreters created, and that the use of nOPV would likely imply lower risk (or ideally no risk) of creating new iVDPV excreters. Thus, although the GPEI and countries continue to shift to the use of nOPV2 for outbreak response (and potentially nOPV1 and/or nOPV3 in the future), we model the situation for which PAVDs would have the largest upper bound of INBs as the base case. The potential reintroduction of poliovirus transmission by iVDPV excreters that exist at the start of the time horizon due to the use of OPV for outbreak responses implies potential benefits of PAVDs. The use of PAVDs may influence whether iVDPV excreters clear the poliovirus infection before potential reintroduction occurs, and thus reduce the risks posed by iVDPVs. If reintroductions of iVDPVs result in widespread transmission, the progeny VDPVs may get detected and classified as ambiguous VDPVs (aVDPVs) or cVDPVs depending on the nature of their detection and their extent of reversion and spread. Currently, paralytic cases in PID patients caused by iVDPVs (iVAPP cases) do not appear in the global counts of cVDPVs,^51^ but instead appear in separate reports.^3,13,20^

### Economic analysis framing, cost and valuation inputs

Early clinical studies using a single antiviral compound identified the development of resistance to PAVDs as a key issue,^46^ and motivated the development of a second compound with a different mechanism of action for combination therapy. We therefore assume that future clinical protocols for treatment of iVDPV excreters will involve treatment with the two separate compounds. Similar to prior analyses,^19,43^ we provide an upper bound for the financial resources that would still lead to positive estimate of the INBs. Thus, recognizing the current state of PAVD development, for which continued investments could lead to the availability of 2 compounds for use as a combined product, we focus on estimating the INBs while ignoring PAVD research and development costs and any costs associated with the identification, screening, and treatment of iVDPV excreters with PAVDs. Although we do not attempt to quantify them, PAVD research and development costs may prove quite substantial, as expected for typical drug development efforts. These costs should be captured in health economic analyses that include total social costs (i.e., all costs, independent of who pays them). For typical drug development processes, the manufacturers would expect to recover the costs of research, development, production, distribution, regulatory, and stewardship, and these costs would factor into pricing. Thus, the resulting INB estimate represents an upper bound of the INBs, such that as long as the total costs of all of the activities required to develop and use the PAVDs falls below that bound, we might expect a positive INB.

For this analysis, we rely on published cost inputs, methods, and assumptions and focus on total social costs using a societal perspective.^38,48^ The integrated model includes numerous cost assumptions for polio-related interventions, including immunization, outbreak response, surveillance to identify poliovirus transmission in populations, as well as treating polio cases and societal costs associated with productivity losses.^48^ The benefit of reducing (or eliminating) the reintroduction risks posed by iVDPV excreters translate to potentially fewer outbreaks (and thus avoided cases, treatment, and productivity costs), avoided outbreak response costs, and increased chances of a successful polio eradication endgame.^19,43^ We use updated costs assumptions developed for the integrated model that use 2019 US dollars (US$2019).^48^. Consideration of the potential extended public health and/or clinical benefits of PAVDs beyond the control of iVDPVs fall outside the scope of this manuscript.

### Policy and scenario assumptions

As demonstrated in prior studies, the global policies related to OPV cessation play a significant role in the value of PAVDs.^20,43,47^ Although OPV2 cessation did not succeed within the first 7 years,^52^ GPEI still plans to globally coordinate bOPV, achieve OPV2 cessation, and certify these achievements by 2027.^35^ Despite uncertainty about when and whether OPV cessation will occur, similar to a recent analysis,^37^ we take OPV cessation as a given for this analysis. In this regard, the key assumptions relate to when bOPV cessation will occur, for which we assume WPV1 elimination in 2023, and bOPV cessation coordinated in early 2027. We implemented these assumptions in the prospective model as occurring on May 1, 2027, for the base case scenario.^37^ During the interim between WPV1 elimination and bOPV cessation, we assume that GPEI will undertake a process that will include global certification of the eradication of WPV1 and all of the planning required for coordinated bOPV cessation.

We assume that the development timeline for PAVDs could lead to their availability for treatment of iVDPVs as early as bOPV cessation. We explore the potential impact of PAVDs by considering the same two different levels of drug effectiveness used in prior modeling (i.e., 40% for a lower bound and 90% for an upper bound).^43^ For comparison with a prior study, we consider three main PAVD use scenarios: (1) a base case of no PAVD use (base case), (2) passive PAVD use, which assumes screening identifies 50% of iVDPV excreters with iVAPP and treats them with PAVDs, and (3) active PAVD use, which assumes screening identifies 90% of all iVDPV excreters (including asymptomatic ones) and treats them with PAVDs.^43^ We began our analysis with the *no PAVD base case* with bOPV cessation on May 1, 2027, and with the bounding scenarios of reflecting the lower and upper bounds of potential impacts of PAVD use: (i) *passive PAVD 40% effectiveness*, which assumes 40% PAVD effectiveness and passive identification of iVDPV excreters, and (ii) *active PAVD 90% effectiveness*, which assumes 90% PAVD effectiveness and active identification of iVDPV excreters. We evaluated the need for additional scenarios using mOPV2 following the analysis of these bounding scenarios.

Recognizing GPEI shift toward the use of nOPV2 for outbreak response,^24,25^ as well as remaining uncertainty about its actual performance (with active research continuing to increase the evidence base), we also explored the potential trajectories for the polio endgame without PAVDs using nOPV2 instead of mOPV2 for outbreak response for bounding case assumptions on the performance of nOPV2 developed in detail elsewhere.^37^ The nOPV2 bounding case assumptions range from the *best nOPV* (defined as the same effectiveness as mOPV2, no VAPP, and no reversion to neurovirulence) and *worst nOPV* (defined as less effective than mOPV2 and some reversion to neurovirulence, with the potential to create new iVDPV2 excreters).^37^ For these analyses, we assumed that after bOPV cessation in 2027, any outbreak response would also use homotypic nOPV for types 1 and 3^53^ (e.g., for the *best nOPV* scenario, this means nOPV1, nOPV2, and nOPV3 with all of the best attributes defined as the same effectiveness as homotypic mOPV, no VAPP, and no reversion to neurovirulence).

Table 1 lists key policy assumptions relevant to iVDPVs and potential PAVD benefits, as well as PAVD-specific assumptions for this analysis. The top three rows of Table 1 indicate assumptions constant across all modeled scenarios, while the bottom three rows list the assumptions (and their value ranges) that vary for different scenarios. Specifically, for all considered scenarios we assume that: (i) OPV will be allowed for use during outbreak response in perpetuity after the type-specific OPV cessation, (ii) IPV use in routine immunization will continue in perpetuity after the cessation of last OPV serotype, (iii) bOPV cessation will occur in 2027, (iv) PAVD introduction will occur in 2027, and (v) the PAVD use and effectiveness will vary between the scenarios.

We implement the model in JAVA^™^ in the integrated development environment Eclipse^™^, and perform 100 stochastic iterations with a fixed set of random number seeds and initial conditions over an analytical time horizon of 2022–2035 for each scenario. Using the same 100 sets of inputs controls for parameter uncertainty in the simulation. We did not further explore parameter uncertainty because based on prior experience we did not anticipate substantial changes in the results of this analysis.^54,55^ We continue to learn from reflection on prior modeling and look back analyses to inform our prospective integrated modeling.^52,56^

## 3. Results

**Figure 2:**
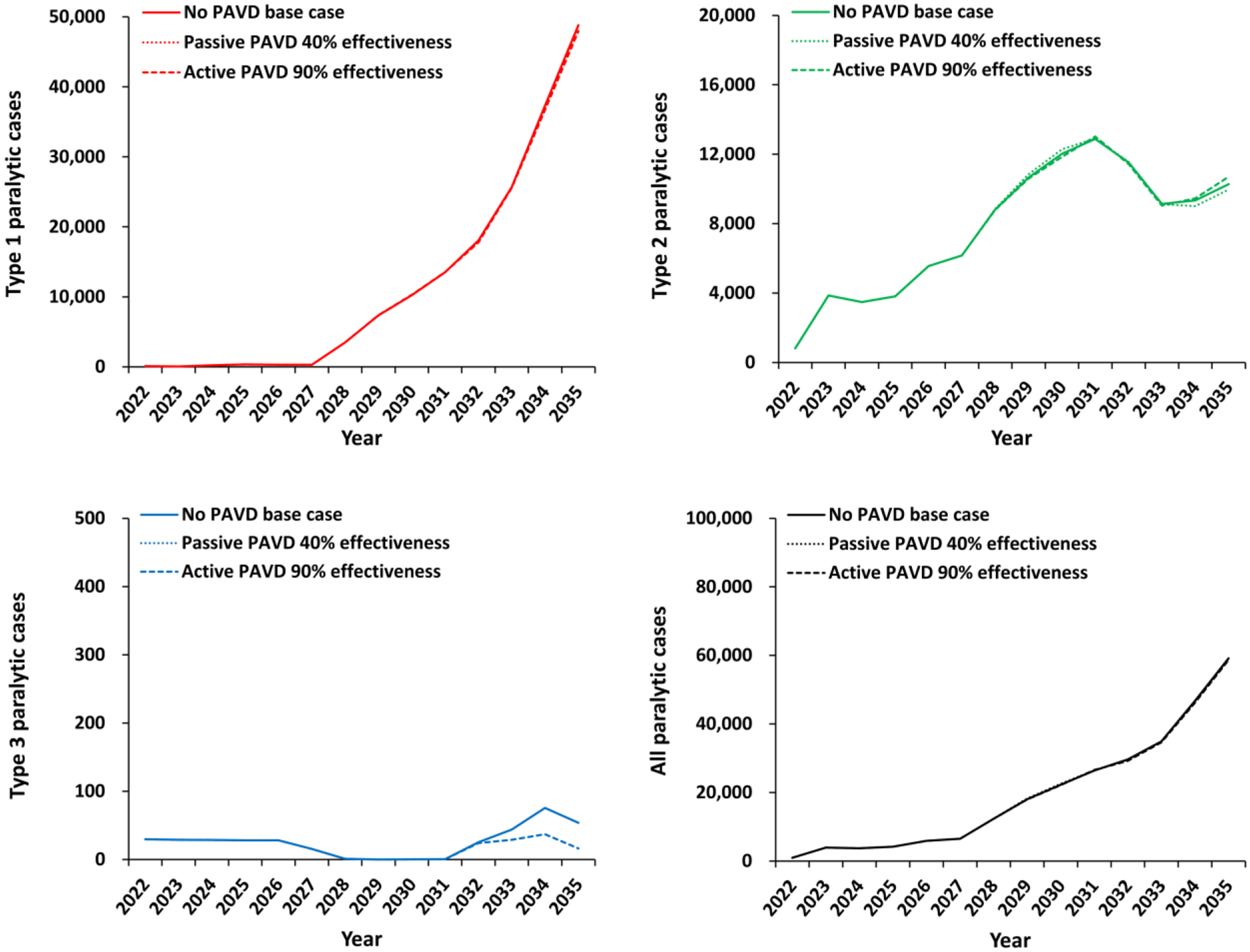
Expected global number of polio cases by year for 100 stochastic iterations of the different PAVD policy choices for the period 2022–2035. **Abbreviations:** PAVD, polio antiviral drug

Figure 2 (panels a-c) shows the expected value of annual paralytic polio cases for the time horizon (2022–2035) caused by type 1, 2, and 3 polioviruses, respectively, as well as the total number of cases (panel d). Figure 2 includes the results without PAVDs (i.e., *no PAVD base case*), as well as the lower and upper bound scenarios (i.e., *passive PAVD 40% effectiveness* and *active PAVD 90% effectiveness*, respectively). Results shown in Figure 2 represent cumulative paralytic cases and do not distinguish paralysis cases caused by WPVs from those due to VDPVs. Red, green, and blue colored lines in panels a-c of Figure 2 represent poliovirus types 1, 2, and 3, respectively, with no other significance. We discuss the results in the context of some type-specific observations, but evaluate the INBs using the overall impacts of PAVDs based on the combined experience for all types of polioviruses, with panel d of Figure 2 showing these combined results. Supplemental Figures S1–S3 show the expected value curves shown as bold black lines in the background of the 100 individual model outputs for each of the 3 modeled scenarios.

Type 1 polioviruses (Figure 2, panel a) show a biphasic behavior for the 2022–2035 time horizon as a result of model inputs used to reflect the stated plans and practices of the GPEI.^35^ Specifically, in the model, WPV1 transmission stops in 2023 and the total number of type 1 paralytic polio cases remains low as long as bOPV use continues. However, with bOPV cessation anticipated in 2027,^35^ in the background of less than ideal global immunization coverage, the model shows a rapid and significant rise in type 1 paralytic polio cases as a result of increasing type 1 cVDPV cases (cVDPV1s).^37^ This rapid increase in paralytic cases occurs due to the high transmissibility and neurovirulence of type 1 polioviruses, with the expected value increased by worst case iterations in which transmission occurs in countries with high transmission potential and/or low coverage.^57^ Although countries and the GPEI could potentially manage bOPV cessation to minimize and potentially eliminate the risks of cVDPV1 cases,^58,59^ current GPEI plans appear poised to repeat the experience of insufficient population immunity to transmission prior to OPV cessation that occurred with OPV2 cessation.^37^ Table 2 summarizes the results from the 100 iterations for the number of iVDPV introductions by type, with 53 total expected iVDPV introductions on average for the 100 iterations the *No PAVD* scenario, and Table 3 summarizes the results for the numbers of cases by type, with 162 total expected cases on average for the 100 iterations of the *No PAVD* scenario. With respect to PAVDs, the results show that the *passive PAVD 40% effectiveness* scenario (Figure 2, panel a, dotted line) stops less than one expected iVDPV related introduction (Table 2, out of 53 expected to occur during the time horizon). However, as shown in Table 3, the *passive PAVD 40% effectiveness* scenario prevents 162 expected polio cases. The *active PAVD 90% effectiveness* scenario (Figure 2, panel a, dashed line) prevents 23 expected iVDPV related introductions (Table 2), which in turn prevents 1,586 expected polio cases (Table 3). PAVDs have a high initial impact on type 1 cases due to assumed immediate PAVDs availability after bOPV cessation in 2027 and before the rapid increase in cVDPV1 cases, However, this short-lived effect quickly disappears as the cVDPV1 cases increase as a result of insufficient population immunity to type 1 poliovirus transmission at the time of cessation^59^ and dominate the annual expected cases shown in Figure 2.^37^

Type 2 polioviruses (Figure 2, panel b) show different behavior because of current cVDPV2 transmission dynamics. The model assumes the introduction of PAVDs in 2027, but the continued use of mOPV2 outbreak response in the background of already high cVDPV2 transmission dampens the effect of PAVDs. Figure 2 shows a counterintuitive increase of type 2 cases after 2032 with the *active PAVD 90% effectiveness* scenario. Although PAVD use stops many expected iVDPV-related introductions expected during the time horizon (i.e., for 14 out of 21 for the 100 iterations in Table 2), the change in the transmission dynamics that occurs in the still affected stochastic iterations effectively shifts the overall expected timing of outbreak response to later times. Delayed (longer) outbreak response times due to later detection increases the expected number of type 2 cases compared with the *no PAVD base case*. Similar to type 1 polioviruses, the results show a very small overall expected impact of PAVDs on the trajectory of type 2 cases, because the current trajectory shows no progress towards elimination within the time horizon. Upon observation of these results, we did not see value in performing any additional analyses, given that even highly effective PAVDs administered to nearly all iVDPV excreters using the most optimistic assumptions for PAVD performance and iVDPV excreter identification will not substantially improve expected polio endgame outcomes.

Type 3 polioviruses (Figure 2, panel c) are negligible compared to types 1 and 2, and the type 3 results do not play a meaningful role in the INBs of PAVDs over the time horizon. While active screening and the use of high effectiveness PAVDs have the potential for positive impact on individuals receiving the treatment, the expected overall effect on global poliovirus transmission remains limited (Figure 2, panel d).

Given the limited overall effect expected with the current polio endgame, we did not perform other alternative analyses, which by design would represent worse options. Tables 2 and 3 provide summary statistics for the number of iVDPV related poliovirus introductions and poliovirus cases, respectively, for the 100 stochastic iterations summed over the entire time horizon.

Table 4 summarizes the results of the incremental economic analyses for alternative PAVD use bounding scenarios compared to the *no PAVD base case* by World Bank income levels and the total global INBs over the 14-year time horizon. Compared to the *no PAVD base case*, the lower bound of *passive PAVD 40% effectiveness* leads to expected loss (i.e., an overall decrease in INBs by 2.1 million US$2019), even without accounting for the costs of the PAVD research, development, and costs of identifying the iVDPV excreters to treat or costs of treating them, which would make the overall INBs much worse (i.e., more negative). Comparison of the *no PAVD base case* with the *active PAVD 90% effectiveness* option offers an expected increase in INBs of 59.3 million US$2019, when assuming no additional cost of PAVD development, PID screening, and PAVD production and administration. This suggests that even under the most optimistic assumptions for PAVD performance and iVDPV detections, all costs associated with PAVD use would need to fall below the 60 million US$2019 to make PAVD development an economically viable option based on INB criteria.

Turning to the potential role of using nOPV instead of mOPV for outbreak response, we started with the no PAVD base case. Supplemental Figures S1 and S4–S6 show the results for the no PAVDs base case, no PAVDs best nOPV, and no PAVDs worst nOPV scenarios (for comparison with Figures 2 and S1–S3). For type 1 (panel a comparisons), the use of nOPV1 results in a slightly slower increase in expected paralytic cases over the time horizon, because using nOPV1 for outbreak response instead of mOPV1 after bOPV cessation comes with no (No PAVD best nOPV) or lower (No PAVD worst nOPV) risks of seeding new transmission than using mOPV1. For type 2 (panel b comparisons), replacing mOPV2 with best nOPV2 lowers the overall expected burden of type 2 paralytic disease, but the model still does not predict type 2 elimination.^37^ Table 5 summarizes the expected iVDPV introductions for the no PAVDs base case, no PAVDs best nOPV, and no PAVDs worst nOPV scenarios. Although slowing the increase and lowering the overall burden of disease using nOPV might lead to an assumption of potentially increased benefits of PAVDs, the use of best nOPV perhaps counterintuitively leads to lower expected INBs. The even lower economic favorability of PAVDs with best OPV use for outbreak response occurs due to the reduced number of iVDPV introductions (Table 5), which implies fewer future individuals who might benefit from PAVD use in the context of still increasing global transmission of cVDPVs. If nOPVs perform like the worst nOPV scenario (defined as less effective than mOPV and some reversion to neurovirulence,^26,60^ with the potential to create new iVDPV excreters) then using PAVDs would imply INBs similar to those for mOPV.

## 4. Discussion

While the polio endgame continues to extend beyond the originally expected time horizons, uncertainty remains about the prospects of successful OPV cessation. Although prior health economic analyses suggested some justification for investments in PAVDs,^19,43^ the current polio endgame appears likely to including ongoing OPV use in the foreseeable future. In simulations of the current polio endgame,^37^ the development of PAVDs shows substantially lower expected potential INBs, even with the assumption of continued use of mOPV for outbreak response, for which the continued potential creation of new iVDPV excreters over time would lead to the largest expected potential INBs.

The use of nOPV instead of mOPV for outbreak response^32,61^ could lead to even lower expected INBs for PAVDs because nOPVs by design would reduce the chances of creating new iVDPV excreters compared with Sabin OPVs. Although the selective use of nOPV2 since 2021 provides some insights into performance, and research towards the development of types 1 and 3 novel OPVs continues, the economic case for PAVDs depends on the ability of global efforts to successfully stop all OPV use. We did not formally consider the uncertain impacts of novel vaccines on the economics of PAVDs, primarily because we recognized that the use of nOPV2 would most likely lead to lower INBs independent of uncertainty about its field performance. Thus, we do not expect better results in favor of PAVD use following the potential replacement of Sabin OPV(s) with homotypic nOPV(s).

Enterovirus infections are associated with significant morbidity, but in spite of numerous potential candidates,^62^ no anti-enteroviral drugs have been effectively deployed for polio. The recent flurry of antiviral development for COVID-19 could also potentially accelerate identification of effective antiviral drugs for polio. Interestingly, a known, long-term (>30 years) chronic excreter appears to have stopped excreting polioviruses after COVID-19 infection and Paxlovid^™^ treatment, but uncertainty remains about whether COVID-19 infection or the antiviral drug led to the end of poliovirus infection in this individual.^63^ Generally, the regulatory path to approval of a specific PAVD continues to prove more challenging than might occur with the potential off-label use or repurposing of an existing antiviral or anti-enteroviral drug. The compassionate use of PAVDs for individual iVDPV excreters could potentially continue as part of studies of investigational new drugs, or could come under consideration as orphan drugs (i.e., pharmaceutical products for which insufficient markets exist to support their commercial use). Research and development in PAVDs and related antivirals will likely continue by investors who see a benefit in development of such drugs independent of the polio eradication program.

As with prior applications of the integrated model, this analysis comes with several limitations related to the model structure, available information, and our assumptions, particularly about the initial conditions as of the end of 2021 and expected future policies and actions.^32,38^ Moreover, the results depend on the implicit assumption of unlimited vaccine supplies, although real constraints have impacted GPEI activities for polio vaccines. In addition, the absence of specific data about the clinical effectiveness of PAVDs, and whether or not the PAVDs currently under development could successfully lead to licensure by our assumed timeline represent other implicit uncertainties. Finally, our model inputs assume implementation of current GPEI plans as we understand them for eradication of WPV1, control of cVDPV2, and cessation of bOPV use.^35^ With the evolving landscape of the polio endgame, GPEI policies, plans, and timelines may change, as they have done on multiple occasions over the past two decades. Most importantly, if GPEI and country performance with respect to outbreak response, immunization coverage, and PID surveillance improves significantly over the next few years, investments in PAVDs may have a bigger impact on the polio endgame that shown in our current study.

In a world with unlimited resources, development of PAVDs and implementation of active screening programs for PIDs and iVDPV would offer some value. However, in the context of limited global resources for polio eradication and the need for resource prioritization, our analysis suggests small INBs of further investments in PAVDs and screening for iVDPVs. Faced with ongoing challenges with control of type 2 cVDPVs and an anticipated challenge with cocirculating type 1 cVDPVs,^64^ expected to increase after the proposed bOPV cessation in 2027,^37^ significant financial and human resources are needed to boost and maintain population immunity to stop and prevent poliovirus transmission. With cVDPVs posing a significantly larger threat to the polio endgame in the foreseeable future, we expect interventions that target iVDPVs will play a smaller role in the polio endgame.

## Supplementary Material

Supplemental Material

## Figures and Tables

**Table 1:** The policy assumptions relevant to PAVD use and modeled scenarios

Model assumption	Base case	Alternatives
Homotypic OPV allowed for oSIAs after OPV cessation of each type	entire time horizon	
IPV use in routine immunization after the cessation of last OPV serotype	entire time horizon	
bOPV cessation time	May 1, 2027	
PAVD introduction time	NA	bOPV cessation
PAVD effectiveness	0%	40%, 90%
PAVD use approach	no PAVD	passive*, active**

Notes:

* screening identifies 50% of iVDPV excreters with iVAPP and treats them with PAVDs with 40% effectiveness

** screening identifies 90% of all iVDPV excreters and treats them with PAVDs with 90% effectiveness

**Abbreviations:**bOPV, bivalent OPV (types 1 and 3); IPV, inactivated poliovirus vaccine; OPV, oral poliovirus vaccine; oSIAs, outbreak supplemental immunization activities; PAVD, polio antiviral drug

**Table 2:** Estimated expected value (median) and [range] of global iVDPV-related introductions for 100 stochastic iterations for the time horizon of 2022–2035 for the scenarios modeled and by type.

	Expected value of iVDPV-related introductions (median) [range]			
Scenario	iVDPV1	iVDPV2	iVDPV3	Total
*No PAVD base case*	22 (21) [9 – 46]	21 (21) [2 – 53]	10 (10) [1 – 22]	53 (53) [28 – 81]
*Passive PAVD, 40% effectiveness*	22 (21) [9 – 46]	21 (20) [1 – 53]	10 (10) [1 – 22]	53 (52) [28 – 80]
*Active PAVD, 90% effectiveness*	14 (13) [5 – 31]	7 (6) [0 – 17]	10 (9) [1 – 22]	30 (29) [15 – 52]

**Abbreviations:**iVDPV(1,2,3), immunodeficiency-associated vaccine-derived poliovirus (type 1, 2, 3); PAVD, polio antiviral drug

**Table 3:** Estimated expected value (median) and [range] of poliovirus cases in 100 stochastic iterations for 2022–2035 for the scenarios modeled.

Scenario	Type 1 cases (median) [range]	Type 2 cases (median) [range]	Type 3 cases (median) [range]	Total cases (median) [range]
*No PAVD base case*	165,740 (163,625) [36,166 – 291,839]	108,374 (110,865) [32,488 – 170,484]	653 (450) [446 – 9,717]	274,767 (270,821) [122,670 – 423,569]
*Passive PAVD 40% effectiveness*	165,698 (162,030) [36,166 – 291,839]	108,254 (110,865) [32,488 – 170,485]	653 (450) [446 – 9,717]	274,605 (270,821) [122,670 – 423,569]
*Active PAVD 90% effectiveness*	164,008 (160,430) [36,166 – 297,337]	108,612 (112,202) [32,487 – 170,485]	560 (450) [446 – 8,252]	273,181 (270,809) [115,604 – 418,228]

**Abbreviations:**PAVD, polio antiviral drug

**Table 4.** Incremental economic analysis estimates (US$2019) for different immunization options for different policy options by World Bank Income Levels (2022–2035)

Vaccine policy	Base case paralytic cases	Policy paralytic cases	Cases prevented	Base case vaccine costs (millions)	Policy vaccine costs (millions)	Incremental financial costs* (millions)	Incremental net benefits* (INBs, millions)
*Passive PAVD 40% effectiveness vs. no PAVD base case*							
LI	97,492	97,493	−1	4,920.9	4,920.9	0.0	0.0
LMI	169,377	169,234	143	11,041.7	11,045.4	3.7	−1.2
UMI	7,853	7,831	22	13,417.4	13,417.3	−0.1	2.6
HI	45	47	−2	18,925.7	18,927.1	1.3	−3.5
Total	**274,767**	**274,605**	**162**	**48,305.6**	**48,310.6**	**4.9**	−**2.1**
*Active PAVD 90% effectiveness vs. no PAVD base case*							
LI	97,492	96,692	800	4,920.9	4,911.9	−9.0	15.6
LMI	169,377	168,592	785	11,041.7	11,021.8	−19.8	41.8
UMI	7,853	7,851	2	13,417.4	13,415.7	−1.7	2.2
HI	45	45	0	18,925.7	18,926.0	0.3	−0.3
Total	**274,767**	**273,181**	**1,586**	**48,305.6**	**48,275.4**	−**30.3**	**59.3**

Notes:

* includes treatment costs of paralytic case, does not include PAVD policy related costs

**Abbreviations:**HI, high income; LI, low-income; LMI, lower middle-income; PAVD, polio antiviral drug; UMI, upper middle-income, US$2019, 2019 United States dollars

**Table 5:** Estimated expected value (median) and [range] of global iVDPV-related introductions by type for 100 stochastic iterations for the time horizon of 2022–2035 for the scenarios modeled with No PAVDs assuming difference vaccines used for outbreak response.

	Expected value of iVDPV-related introductions (median) [range]			
Scenario	iVDPV1	iVDPV2	iVDPV3	Total
*No PAVD base case*	22 (21) [9 – 46]	21 (21) [2 – 53]	10 (10) [1 – 22]	53 (53) [28 – 81]
*No PAVD best nOPV*	13 (12) [3 – 27]	1 (0) [0 – 9]	10 (9) [1 – 22]	24 (24) [10 – 45]
*No PAVD worst nOPV*	20 (19) [8 – 40]	25 (26) [2 – 49]	11 (10) [1 – 35]	55 (56) [29 – 81]

## Social Media Content:

Polio antiviral drugs could play an important role in the polio endgame, their expected economic benefits continue to drop with delays in polio eradication and continued use of oral poliovirus vaccines.

The incremental net benefits of investments in development and deployment of polio antiviral drugs are small, and the limited global resources are better spent on routine and supplemental immunization activities.

## Highlights:

- While polio antiviral drugs could play an important role in the polio endgame, their expected economic benefits continue to drop with delays in polio eradication and continued use of oral poliovirus vaccines.
- The incremental net benefits of investments in polio antiviral drug development and screening for immunodeficiency-related circulating polioviruses are small.
- Limited global resources are better spent on increasing global population immunity to polioviruses to stop and prevent poliovirus transmission.
