# Supplementary material for: Health economic analysis of antiviral drugs in the global polio eradication endgame
Source: Med Decis Making. Author manuscript; Available in PMC 2023 Nov 27. (PMC10680042; doi:10.1177/0272989X231191127)
Supplement: Supplemental Material [file NIHMS1946326-supplement-Supplemental_Material.docx]

**Supplement for “Health economic analysis of antiviral drugs in the global polio eradication endgame”**

The integrated model includes a large number of inputs,^1-4^ summarized in Supplemental Table S1. Part (a) summarizes the distribution of the global population by routine immunization policy. Part (b) lists the differential equation-based (DEB) model inputs that remain constant from analysis to analysis. Part (c) provides the model inputs for the recent immunity states by poliovirus type. Part (d) gives average values for model inputs that vary among the 720 model subpopulations. Part (e) shows key policy model inputs and assumptions made related to disruptions that occurred due to the COVID-19 pandemic.

Supplemental Figures S1-S3 show the trajectories of the 100 individual iterations of the model for the specific scenarios, with the bold black trajectory showing the expected value. The black curves shown in Supplemental Figures S1-S3 essentially overlap in Figure 2 in the main paper for panels a, b, and d. Supplemental Figure S4 shows the expected values of the *No PAVD base case* and how the trajectories of expected incidence change by type when nOPV replaces mOPV for outbreak response considering the two bounding sets of characteristics of *best nOPV* and *worst nOPV*.

**Supplemental Table S1:**

**(a) Distribution of the global population into epidemiological blocks in the global model by 2019 World Bank income level^5^** **and polio vaccine use.**

| **Income level** | **Polio vaccine use** | | | **Total blocks** |
| --- | --- | --- | --- | --- |
|  | **OPV+IPV** | **IPV/OPV** | **IPV-only** |  |
| **LI** | 6 | 0 | 0 | 6 |
| **LMI** | 27 | 1 | 0 | 28 |
| **UMI** | 19 | 7 | 1 | 27 |
| **HI** | 0 | 0 | 11 | 11 |
| **Total blocks** | 52 | 8 | 12 | 72 |

**Acronyms:** HI, high-income; IPV, inactivated poliovirus vaccine; LMI, lower middle-income; LI, low-income; OPV, oral poliovirus vaccine; UMI, upper middle-income)

1. **Constant global differential equation-based poliovirus transmission model inputs**

| **Model input (unit) (*symbol*)** | **Value(s)** |
| --- | --- |
| Demographic data for all situations | Time series 1950-2099 |
| Age groups | 0-2, 3–11 months; 1–4, 5-9, 10–14, 15-39; ≥ 40 years |
| Number of immunity states (*ni)* | 8 |
| Number of waning stages (*nw*) | 5 |
| Number of infection stages (*r+s*)  - number of latent stages I (stages 0,1)  - number of infectious stages (*s*) (stages 2,3,4,5) | 6  2  4 |
| Number of OPV reversion stages (*h*)  - Sabin OPV (stage 0)  - OPV-related (stages 1-18)  - fully-reverted poliovirus (assumed equivalent to homotypic WPV) (stage 19) | 20  1  18  1 |
| Transition rates (days)  - average time to full susceptibility for maternally immune infants (*ρ_MI_*)  - average time to develop IPV immunity after successful dose (*φ*) | 0.25×365  7 |
| Shape of waning function (*z_w_*) | 5 |
| Average time to reach last waning stage (*ρ*, in days) (PV1; PV2; PV3) | 4×365; 4×365; 3×365 |
| Duration of latent period (*ξ^fec^* or *ξ^oro^*, in days) | ~ 3^a^ |
| Relative weight of infection stages compared to average weight over the infectious period (*θ_k_*, *k*=0, …, *r*+*s-1*) (stage 0;1;2;3;4;5) | 0;0;12/17;40/17;12/17;4/17 |
| Average time to reach last OPV reversion stage (*ε*, in days) (for PV1; PV2; PV3) | 620.5; 408; 620.5 |
| Shape of OPV reversion function with respect to:  - R_0_ (*z_r_*)  - ln(PIR) (*z_p_*) | 1  2.5 |
| Paralysis-to-infection ratios (PV1; PV2; PV3)  - for fully susceptible individuals infected with FRPV (*PIR_h_*_-1_)  - for fully susceptible individuals infected with OPV (*PIR*_0_) | 0.005; 0.0005; 0.001  7.4 ×10^-8^; 6.2 ×10^-7^; 1.3 ×10^-6^ |
| Relative PIR for maternally immune compared to fully susceptible (*RPIR*_MI_) | 0.5 |
| Ratio of R_0­­_ by serotype in the same setting (PV1:PV2:PV3) | 1:0.9:0.75 |
| Relative R_0_ of OPV vs. FRPV (*τ_0_*) (PV1; PV2; PV3) | 0.37;0.55;0.25 |
| Exportation threshold (E*, i.e., cumulative effective infections needed to trigger a potential exportation from a subpopulation)  - LI and LMI countries  - UMI and HI countries | 125,000  25,000 |
| Proportion of virus exportations  - within the same block  - in another block within the same preferentially mixing area (PMA)  - outside of the PMA | 0.960  0.035  0.005 |
| Relative coverage with birth dose compared to non-birth RI coverage with 3 doses (*relbd*)  - blocks in LI and LMI countries that use OPV+IPV at T_0_  - all other blocks | 0.5  0 |
| Number of pSIAs  - LI, LMI, and UMI countries  - HI countries | Time series  NA |
| Average per-dose take rate for IPV (*tr^IPV^*)  - LI and LMI countries  - UMI countries  - HI countries | 0.63  0.70  0.75 |
| Average time from introduction to potential detection (days) | 10 |
| Time from outbreak detection until the first oSIA (days)  - no ongoing outbreak response in block, before OPV2 cessation  - no ongoing outbreak response in block, after OPV2 cessation  - outbreak response already ongoing in block | 60  45  30 |
| Duration of each oSIA (days) | 5 |
| Number of oSIA rounds  - before OPV2 cessation  - after OPV2 cessation | 3  2 |
| Time interval between oSIA rounds (days) | 30 |
| Effective infectious proportion below which we assume 0 force-of-infection (transmission threshold *EPI**) | 5/1,000,000 |

**Abbreviations:** FRPV = fully-reverted poliovirus; HI, high-income; IPV, inactivated poliovirus vaccine; LI, low-income; LMI, lower middle-income; NA, not applicable; OPV, oral poliovirus vaccine; OPV2, serotype-2 OPV; oSIA, outbreak response SIA; PIR = paralysis-to-infection ratio; PMA, preferentially mixing area; PV(1,2,3) = poliovirus (type 1, 2, or 3, respectively); R_0_, average annual basic reproduction number; RI, routine immunization; SIA, supplemental immunization activity; T_0_, beginning of analytical time horizon (i.e., January 1, 2019); UMI, upper middle-income; WPV(1,2,3) = wild poliovirus (type 1, 2, or 3, respectively)

**Notes:** ^a^ Mean estimates obtained from experts and used in the model for the different immunity states, serotypes, and excretion modes vary between 2.85 and 3.37 days

1. **Model inputs for immunity states**

| **Immunity state** |  | **Fully susceptible** | **Maternally immune** | **1 successful IPV** | **2 successful IPV** | **≥ 3 successful IPV** | **1 LPV infection** | **≥ 2 LPV infections** | **IPV and LPV** |
| --- | --- | --- | --- | --- | --- | --- | --- | --- | --- |
| Relative susceptibility (*σ*) of recent immunity states for: | PV1  PV2  PV3 | 1.0  1.0  1.0 | 0.78  0.79  0.77 | 0.91  0.92  0.90 | 0.80  0.80  0.79 | 0.72  0.72  0.71 | 0.42  0.43  0.41 | 0.21  0.22  0.20 | 0.21  0.22  0.20 |
| Duration of fecal infectiousness (*γ^fec^*, in days) of recent immunity states for: | PV1  PV2  PV3 | 28.0  27.8  28.3 | 24.6  24.6  24.6 | 24.5  24.4  24.7 | 21.1  20.8  21.3 | 18.0  17.7  18.2 | 11.6  10.5  10.5 | 10.1  8.9  8.9 | 10.1  8.9  8.9 |
| Relative fecal infectiousness (*π^fec^*) of recent immunity states for: | PV1  PV2  PV3 | 1.0  1.0  1.0 | 0.96  0.96  0.95 | 0.92  0.92  0.91 | 0.70  0.69  0.68 | 0.61  0.59  0.59 | 0.39  0.43  0.43 | 0.20  0.23  0.23 | 0.20  0.23  0.23 |
| Duration of oropharyngeal infectiousness (*γ^oro^*, in days) of recent immunity states |  | 13.4 | 11.9 | 9.9 | 6.6 | 6.1 | 5 | 3.7 | 3.7 |
| Relative oropharyngeal infectiousness (*π^oro^*) of recent immunity states |  | 1.0 | 0.68 | 0.3 | 0.17 | 0.12 | 0.33 | 0.21 | 0.21 |
| Relative susceptibility (*σ*) for last waning stage |  | NA | NA | 1.0 | 1.0 | 1.0 | 0.8 | 0.7 | 0.7 |
| Duration of fecal infectiousness (*γ^fec^*, in days) of last waning stage for: | PV1  PV2  PV3 | NA | NA | 26.6  26.4  26.9 | 25.2  25.0  25.5 | 23.8  23.6  24.1 | 14.0  13.9  14.1 | 11.4  11.4  11.6 | 11.4  11.4  11.6 |
| Relative fecal infectiousness (*π^fec^*) of last waning stage |  | NA | NA | 0.95 | 0.9 | 0.85 | 0.5 | 0.3 | 0.3 |
| Duration of oropharyngeal infectiousness (*γ^oro^*, in days) of last waning stage |  | NA | NA | 11.4 | 6.7 | 6.6 | 6.7 | 4 | 4 |
| Relative oropharyngeal infectiousness (*π^oro^*) of last waning stage |  | NA | NA | 0.43 | 0.25 | 0.13 | 0.5 | 0.3 | 0.3 |

**Abbreviations:** IPV, inactivated poliovirus vaccine; LPV = live poliovirus; NA, not applicable; PV(1,2,3) = poliovirus (type 1, 2, or 3, respectively)

**(d) Average values for global model inputs stratified by World Bank Income Level that vary by block and/or subpopulation (full distributions reported elsewhere)**

| **Input** | **LI** | **LMI** | **UMI** | **HI** |
| --- | --- | --- | --- | --- |
| Basic reproduction number (R_0_) | 10.3 | 9.7 | 7 | 4.9 |
| Age-group preferential mixing strength | 0.36 | 0.36 | 0.44 | 0.39 |
| Proportion of transmissions via the oropharyngeal route | 0.3 | 0.36 | 0.61 | 0.83 |
| RI coverage with 3 or more non-birth doses at T_0_ | 0.77 | 0.83 | 0.94 | 0.95 |
| RI coverage with 1 or 2 non-birth doses at T_0_ | 0.20 | 0.15 | 0.07 | 0.23 |
| True pSIA coverage | 0.76 | 0.86 | 0.93 | NA |
| pSIA repeatedly missed probability | 0.71 | 0.58 | 0.53 | NA |
| tOPV take rate for PV1; PV2; PV3 | 0.44; 0.69; 0.35 | 0.44; 0.68; 0.35 | 0.58; 0.74; 0.48 | 0.52; 0.71; 0.43 |
| mOPV take rate for PV1; PV2; PV3 | 0.59; 0.69; 0.59 | 0.59; 0.69; 0.58 | 0.82; 0.87; 0.77 | 0.73; 0.80; 0.69 |
| bOPV take rate for PV1; PV3 | 0.53; 0.53 | 0.54; 0.54 | 0.72; 0.72 | 0.65; 0.65 |
| IPV start year | 2015.7 | 2015.4 | 2013.3 | 2002.2 |
| Detection threshold 1970-2018 (AFP case-based) | 1.8 | 1.3 | 1.3 | 1.4 |
| Detection threshold 2019-2024 (AFP case-based) | 3.8 | 3.3 | 3.3 | 5 |
| Detection threshold 2025-2027 (sentinel) | 12.5 | 8.9 | 5.5 | 5 |
| Detection threshold 2028-2058 (event-based) | 30 | 20 | 10 | 5 |
| ES start year (if existing) | 2015.3 | 2015.5 | 2019 | 2019.9 |
| ES quality level (if existing) | high to very low | high to very low | high to medium | high to medium |
| ES catchment until 2024 (% of sub-population, if existing) | 2.8 | 0.9 | 0.2 | 0.3 |
| ES catchment 2025-2027 (% of sub-population, if existing) | 2.3 | 0.8 | 0.1 | 0.3 |
| ES catchment 2028-2058 (% of sub-population, if existing) | 0.7 | 0.2 | 0.1 | 0.3 |
| True oSIA coverage pre-OPV2 cessation | 0.77 | 0.76 | 0.72 | 0.9 |
| oSIA repeatedly missed probability pre-OPV2 cessation | 0.73 | 0.74 | 0.77 | 0.5 |
| True oSIA coverage post-OPV2 cessation | 0.76 | 0.86 | 0.93 | 0.89 |
| oSIA repeatedly missed probability post-OPV2 cessation | 0.71 | 0.58 | 0.53 | 0.58 |

**Abbreviations:** AFP, acute flaccid paralysis; ES, environmental surveillance; HI, high-income; bOPV, bivalent OPV; IPV, inactivated poliovirus vaccine; LMI, lower middle-income; LI, low-income; mOPV, monovalent OPV; NA, not applicable; OPV, oral poliovirus vaccine; oSIA, outbreak response SIA; pSIA, planned, preventive SIA; PV(1,2,3) = poliovirus (type 1, 2, or 3, respectively); RI, routine immunization; SIA, supplementary immunization activity; T_0_, beginning of analytical time horizon (i.e., January 1, 2019); tOPV, trivalent OPV; UMI, upper middle-income

**(e) Global model base case policy and COVID-19 pandemic disruption inputs**

| **Model input (unit) (*symbol*)** | **Value(s)** |
| --- | --- |
| Prospective model time horizon | 2022-2036 |
| Minimum years of IPV in RI after last OPV cessation | >15 |
| Years of OPV use for oSIAs after homotypic OPV cessation (PV1; PV2; PV3) | >15 |
| Minimum number of doses of IPV in RI after last OPV cessation | 2 |
| Geographical scope of oSIAs  - before OPV2 cessation  - after homotypic OPV cessation, R_0_ < 10  - after homotypic OPV cessation, R_0_ ≥ 10 | Subpopulation  Subpopulation  Subpopulation + 4 worst performing neighbors |
| Target age groups for oSIAs  - before OPV cessation of the serotype  - after OPV cessation of the serotype | 0-2, 3–11 months; 1–4 years  0-2, 3–11 months; 1–4 years |
| True IPV oSIA coverage in post-OPV era | 0.50 |
| IPV oSIA repeatedly missed probability in post-OPV era | 0.80 |
| Average time between contacts of long-term iVDPV excreters with the general population (days) | 150-600 |
| Reversion stage of iVDPV virus when introduced into general population | 10 |
| Global Poisson rate for release of unreturned OPV in blocks that use OPV at T_0_ (1/year)  - within 1^st^ year post serotype specific cessation  - within 2^nd^ year post serotype specific cessation | 4  1 |
| Poor-performing blocks assumed to be more likely to release unreturned OPV | 1, 3, 5, 7, 8, 13, 32, 34 |
| Weight of unreturned OPV releases by block performance  - poor-performing blocks (1/block)  - other performing blocks (1/block) | 5  1 |
| Global Poisson rate for release from IPV production site (1/year) | 0.2 |
| Global Poisson rate for release from other PEF (1/year) | 0.02 |
| Global Poisson rate for release from PIM or other unintentional release (1/year) | 0.004975 |
| Distribution of PIM other unintentional releases by income level  - LI  - LMI  - UMI  - HI | 0.00  0.01  0.09  0.90 |
| Global Poisson rate for other intentional release (1/year) | 0.000025 |
| Distribution of intentional releases by income level  - LI, LMI, UMI  - HI | 0.5  0.5 |
| Serotype-specific cases since OPV cessation to trigger OPV restart | NA (no OPV restart) |
| Time to restart OPV production after OPV cessation (years)  - if licensed OPV manufacturers continue to produce any OPV serotype(s)  - if licensed OPV manufacturers maintain an mOPV stockpile for the serotype  - if no licensed OPV manufacturers exist for any OPV serotype  - if manufacturers already started one OPV serotype for subsequent serotype(s) | 2  3  7  5 |
| COVID-19 RI reduction start date | March 20, 2020 |
| COVID-19 RI reduction end date | December 31, 2020 |
| COVID-19 change in average RI coverage with 3 or more nonbirth doses  LI  LMI  UMI  HI | 0.77 → 0.67  0.83 → 0.73  0.94 → 0.84  0.95 → 0.85 |
| COVID-19 change in average RI coverage with 1 or 2 nonbirth doses  LI  LMI  UMI  HI | 0.20 → 0.17  0.15 → 0.13  0.07 → 0.06  0.23 → 0.20 |
| COVID-19 SIA reduction start date | March 20, 2020 |
| COVID-19 SIA reduction end date | December 31, 2020 |
| COVID-19 Subpopulation-specific oSIA impact level (SI Level^1^) change in non-endemic countries  before September 1, 2020  after September 1, 2020 | no oSIAs  -1 |
| COVID-19 Subpopulation-specific pSIA change in non-endemic countries | no pSIAs |
| COVID-19 Subpopulation-specific SIA impact level (SI Level ^1^) change in endemic countries  before July 1, 2020  after July 1, 2020 | no SIAs  no change |
| Post COVID-19 pSIA change in non-endemic countries  after December 31, 2020 | ≤1 pSIA per year |
| COVID-19 transmission mixing restriction start date | March 20, 2020 |
| COVID-19 transmission mixing restriction end date | August 31, 2021 |
| COVID-19 transmission exportation threshold (E*) factor increase | 1.5 |
| COVID-19 subpopulation-specific R_0_ decrease  March 20, 2020 – May 31, 2020:  Endemic block  Non-endemic blocks  June 1, 2020 – August 31, 2020  September 1, 2020 – November 30, 2020  December 1, 2020 – February 28, 2021  March 1, 2021 – May 31, 2021  June 1, 2021 – August 31, 2021 | -0.125  -1.00  -1.00  -0.75  -0.50  -0.25  -0.125 |

**Abbreviations:** HI, high income; IPV, inactivated poliovirus vaccine; LI, lower-income; LMI, lower-middle income; LPV = live poliovirus; mOPV, monovalent OPV; OPV, oral poliovirus vaccine; OPV2, serotype-2 OPV; oSIA, outbreak response SIA; PEF, polio essential facility; PIM, potentially infectious material; pSIA, planned, preventive SIA; PV(1,2,3) = poliovirus (type 1, 2, or 3, respectively); R_0_, average annual basic reproduction number; RI, routine immunization; SIA, supplemental immunization activity; T_0_, beginning of analytical time horizon (i.e., January 1, 2019); Abbreviations: UMI; upper-middle income

**Supplemental Figure S1:** 100 modeled iterations of *No PAVD base case* for each poliovirus type and totals for all types, with the corresponding expected values shown in black bold lines.


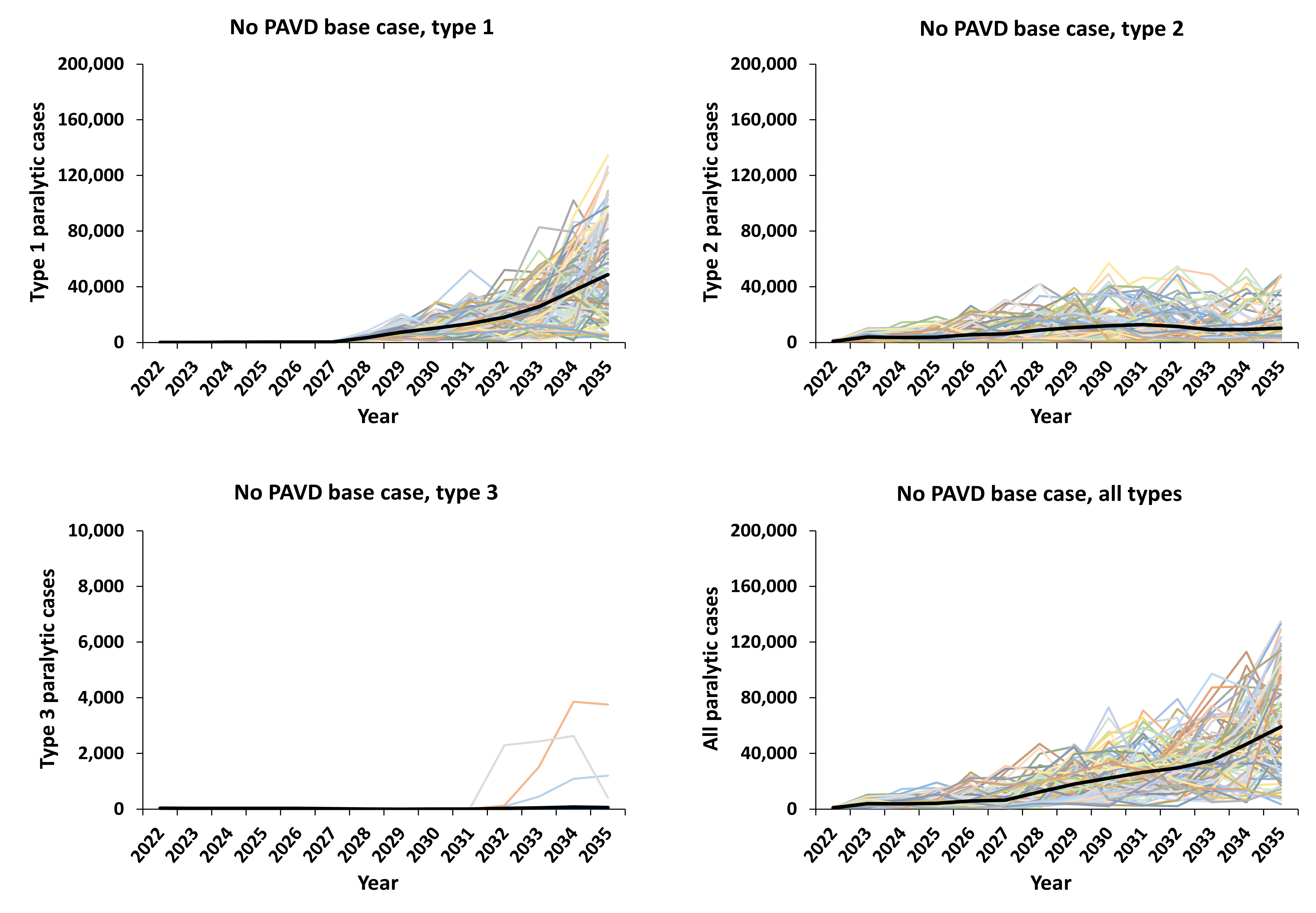


**Abbreviations:** PAVD, polio antiviral drug

**Supplemental Figure S2:** 100 modeled iterations of *Passive PAVD 40% effectiveness* for each poliovirus type and totals for all types, with the corresponding expected values shown in black bold lines.


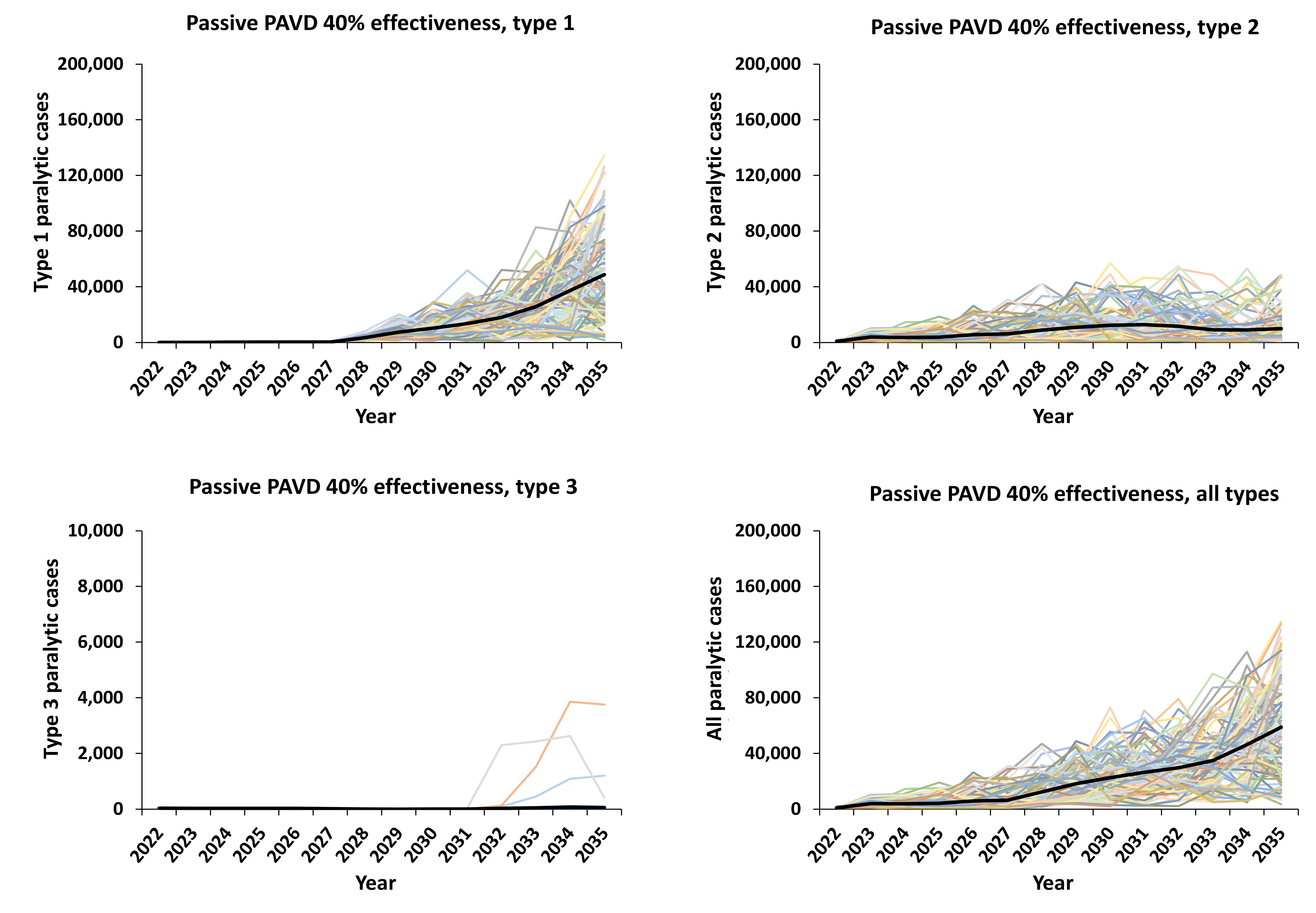


**Abbreviations:** PAVD, polio antiviral drug

**Supplemental Figure S3:** 100 modeled iterations of *Active PAVD 90% effectiveness* for each poliovirus type and totals for all types, with the corresponding expected values shown in black bold lines.


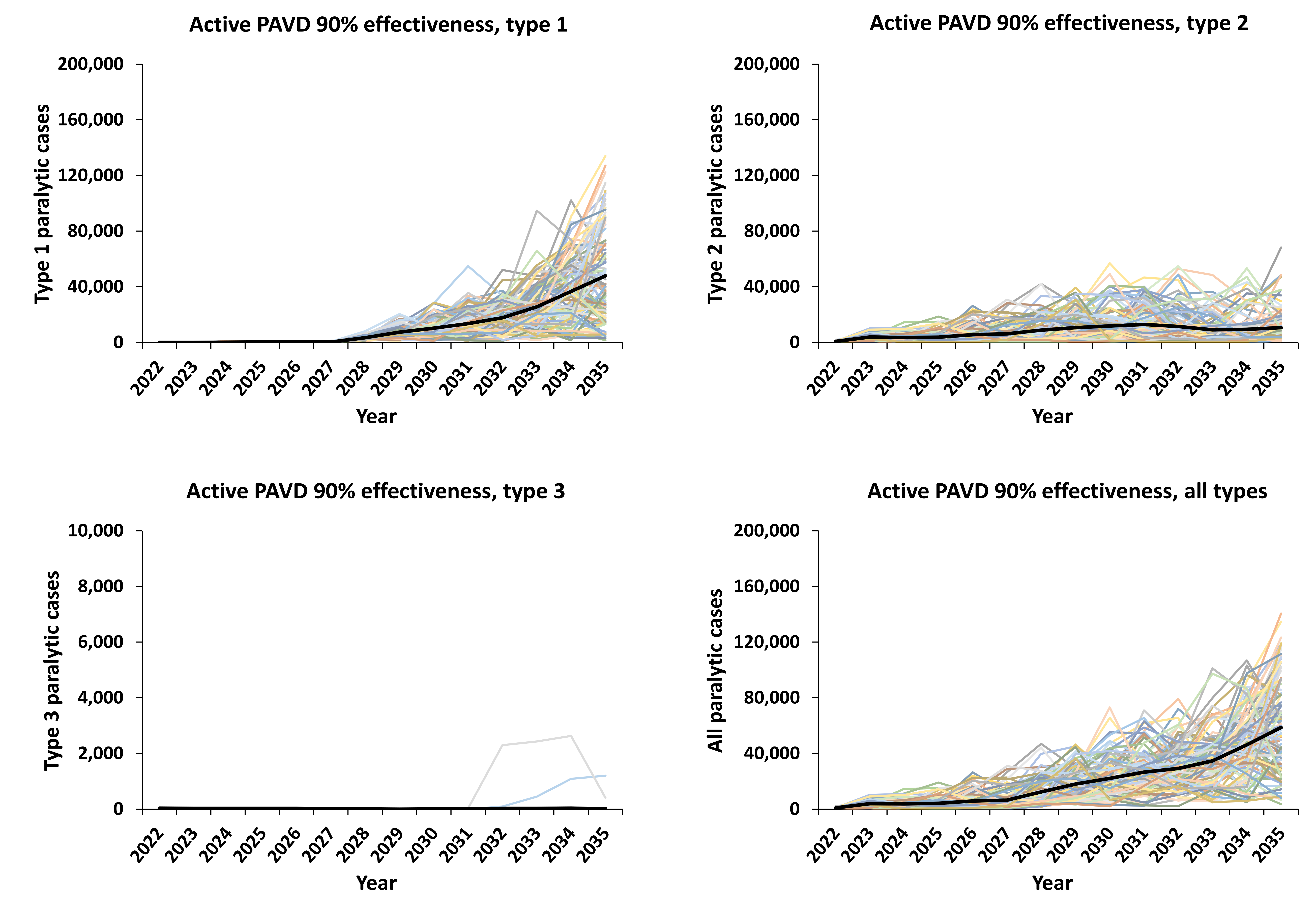


**Abbreviations:** PAVD, polio antiviral drug

**Figure S4:** Expected global number of polio cases by year for 100 stochastic iterations of *no PAVD base case* showing the anticipated reduction in the number of cases if mOPV2 (solid line) is globally replaced by best nOPV (dashed line) or worst nOPV (dash/dotted line). Supplemental Figures S1, S5, and S6 show the expected value curves shown here as bold lines in the background of 100 individual model outputs for each of the 3 modeled scenarios.


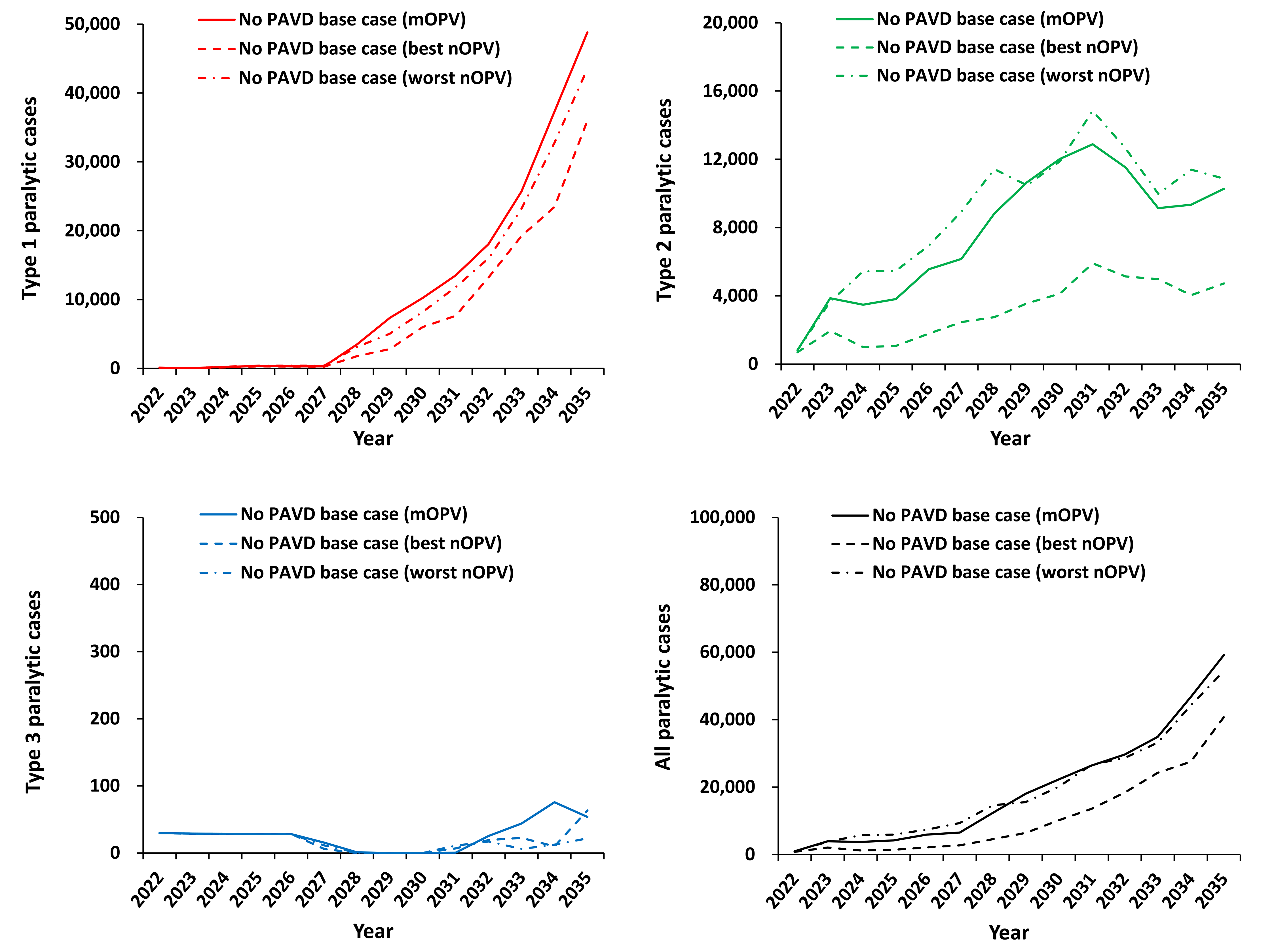


**Abbreviations:** PAVD, polio antiviral drug

**Supplemental Figure S5:** 100 modeled iterations of *No PAVD base case (best nOPV2)* for each poliovirus type and totals for all types, with the corresponding expected values shown in black bold lines.


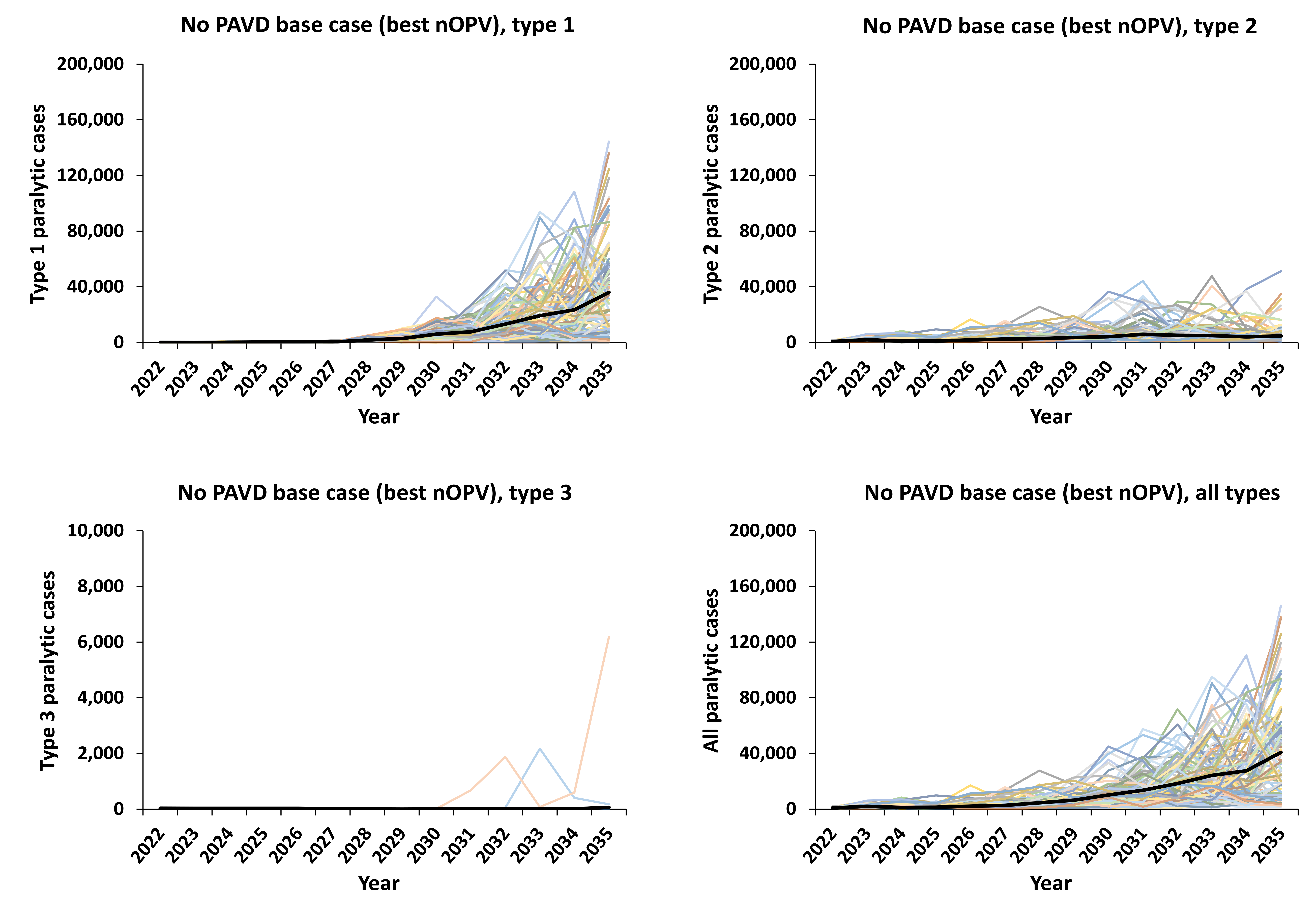


**Abbreviations:** PAVD, polio antiviral drug

**Supplemental Figure S6:** 100 modeled iterations of *No PAVD base case (worst nOPV2)* for each poliovirus type and totals for all types, with the corresponding expected values shown in black bold lines


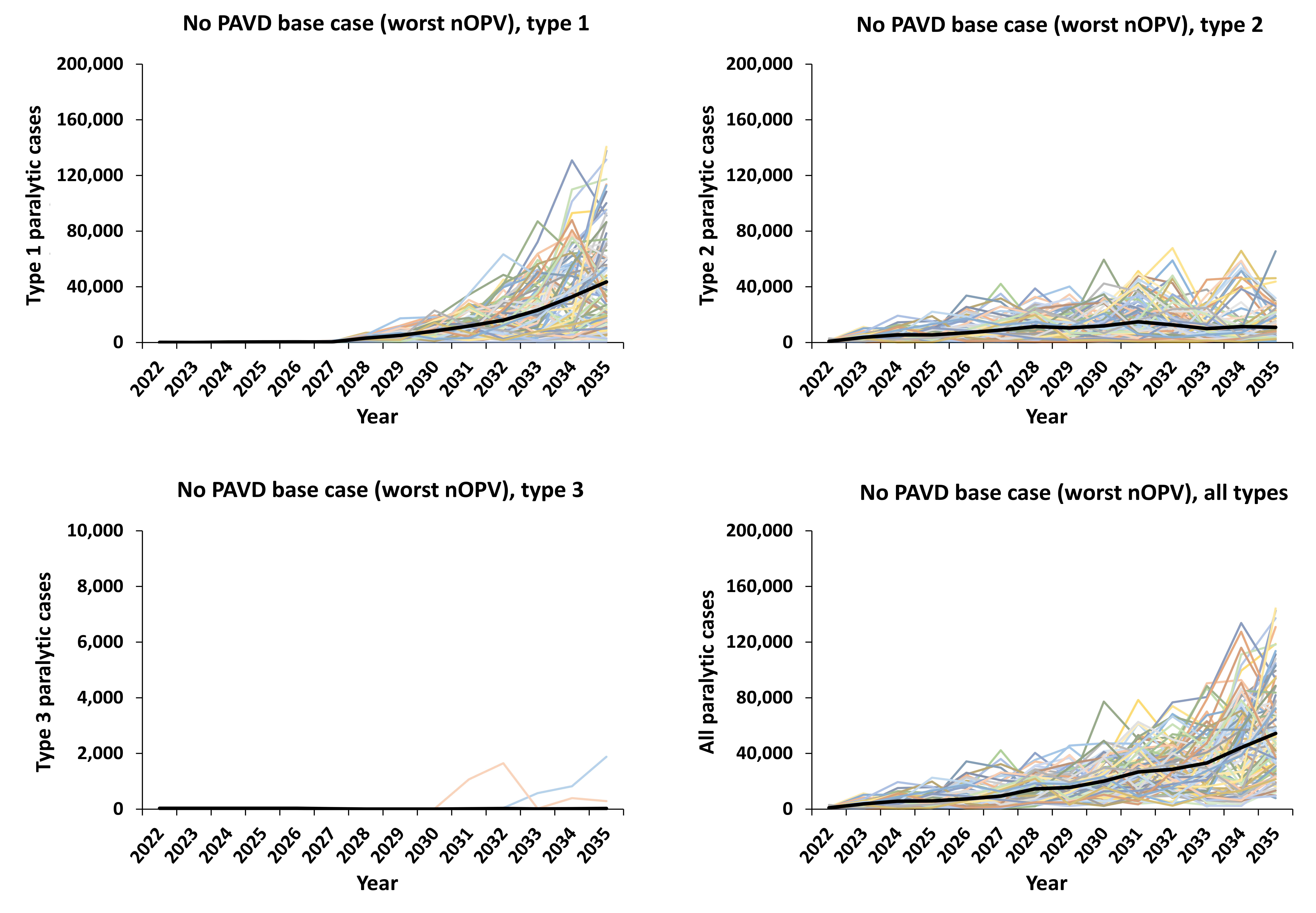


**Abbreviations:** PAVD, polio antiviral drug

**5. References**

1. Kalkowska DA, Wassilak SGF, Cochi SL, et al. Global transmission of live polioviruses: Updated integrated dynamic modeling of the polio endgame. *Risk Anal* 2021; 41: 248-265. 2020/01/22. DOI: 10.1111/risa.13447.

2. Kalkowska DA, Wassilak SGF, Cochi SL, et al. Global transmission of live polioviruses: Updated integrated dynamic modeling of the polio endgame - Technical appendix, <https://www.ncbi.nlm.nih.gov/pmc/articles/PMC7787008/bin/NIHMS1655951-supplement-supplementary_material.pdf> (2021, accessed Jun 8 2023).

3. Kalkowska DA, Pallansch MA, Wilkinson A, et al. Updated characterization of poliovirus outbreak response strategies for 2019-2029: Impacts of the use of novel OPV2 strains. *Risk Anal* 2021; 41: 329-348. DOI: 10.1111/risa.13622.

4. Kalkowska DA, Voorman A, Pallansch MA, et al. The impact of disruptions caused by the COVID-19 pandemic on global polio eradication. *Vaccine* 2023; 41: A12-A18. DOI: <https://doi.org/10.1016/j.vaccine.2021.04.026>.

5. World Bank. World Bank list of economies (June 2019), <http://databank.worldbank.org/data/download/site-content/CLASS.xls> (2019, accessed July 17 2019).
